# Evolution of clonal hematopoiesis on and off lenalidomide maintenance for multiple myeloma

**DOI:** 10.1038/s41375-025-02707-2

**Published:** 2025-07-16

**Authors:** Jennifer H. Cooperrider, Diren Arda Karaoglu, Tadeusz Kubicki, Can Rui Jiang, Ella Postich, Kathryn Shimamoto, Olivia Arnold, Jommel Macaraeg, Aubrianna Ramsland, Anna Pula, Ashwin Kishtagari, Yash Pershad, Taralynn M. Mack, Angela Jones, Alexander G. Bick, Michael Savona, Michael W. Drazer, Dominik Dytfeld, Andrzej Jakubowiak, Benjamin A. Derman, Caner Saygin

**Affiliations:** 1 Section of Hematology/Oncology, Department of Medicine, University of Chicago, Chicago, IL USA; 2 The Department of Hematology and Bone Marrow Transplantation, Poznan University of Medical Sciences, Poznań, Poland; 3 Division of Genetic Medicine, Vanderbilt University Medical Center, Nashville, TN USA; 4 Section of Genetic Medicine, Department of Medicine, University of Chicago, Chicago, IL USA

**Keywords:** Myeloma, Acute myeloid leukaemia

## To The Editor:

Multiple myeloma (MM) accounts for 10% of all blood cancers [1]. Standard of care treatment with autologous stem cell transplantation (ASCT) followed by lenalidomide maintenance improved survival, but MM remains incurable [1]. A diverse therapeutic armamentarium has changed MM into a relapsing-remitting cancer. However, as patients live several years, there has been a steady increase in the frequency of second primary hematologic malignancies (SPHM). In population-based studies, the incidence of SPHM approaches 7% [2]. Several other reports established a relationship between lenalidomide maintenance and therapy-related acute lymphoblastic leukemia (t-ALL) [3, 4]. In randomized phase III DETERMINATION study comparing lenalidomide, bortezomib, dexamethasone with or without ASCT followed by lenalidomide maintenance in frontline treatment of MM, types of SPHM differed between the two arms with high incidence of ALL in non-ASCT arm vs. myeloid neoplasms (MNs) in the ASCT arm [5]. Therefore, SPHM is a devastating life-shortening complication of MM therapies, yet there are no clinically validated tools to identify high-risk individuals and develop personalized prevention strategies.

Clonal hematopoiesis (CH) is a pre-leukemic condition, characterized by the presence of somatic mutations in blood cells of an individual in the absence of hematopoietic malignancy [6]. We and others have shown that CH is a precursor lesion for therapy-related MN and ALL, and these clones can expand under the selective pressure of genotoxic therapy [7–9]. These therapies select for clones harboring mutations in DNA damage repair genes (i.e., *TP53, PPM1D, CHEK2*) since clonal hematopoietic stem cells (HSCs) with these mutations have survival advantage when they regenerate bone marrow after myelotoxic insults. Therefore, most therapy-related MN and ALL cases harbor *TP53* mutations, which are associated with dismal survival outcomes [4, 6]. Preclinical data suggest that continuous lenalidomide therapy leads to expansion of *TP53-*mutant HSCs in mice, but clinical data are limited for clonal evolution of *TP53-*mutant CH in patients receiving long-term lenalidomide maintenance [10]. Whether longitudinal assessment of preleukemic clones can identify MM patients at risk for SPHM has not been studied. We hypothesize that long-term lenalidomide therapy drives progression of high-risk CH in MM, and evaluation of clonal dynamics over time predicts risk for SPHM. We tested this hypothesis by studying prospectively collected hematopoietic samples from two informative MM clinical trials focusing on lenalidomide (R) vs. lenalidomide, carfilzomib, dexamethasone (KRd) maintenance (ATLAS trial [11]), and discontinuation of lenalidomide in patients with measurable residual disease (MRD)-negative remission from MM (MRD2STOP trial [12]). We showed that lenalidomide therapy leads to progression of *TP53-*mutant CH, which precedes t-MN and t-ALL, while discontinuation of lenalidomide stabilizes CH and may lead to regression of high-risk clones.

ATLAS is a randomized phase III study comparing post-ASCT treatment with R vs. KRd maintenance [11]. MRD2STOP is a single institution trial in which patients who completed at least one year of maintenance therapy and achieved MRD negativity at 10^−6^ discontinued lenalidomide [12]. Patients with stored serial samples who provided written informed consent for exploratory analyses were included. DNA was extracted from CD138-negative bone marrow or blood samples of participants at baseline, and serially for two years. Patients had no morphologic or flow cytometric evidence of MM at all time points, excluding any possibility of malignant plasma cell contamination at the detection limit (0.5%) of our CH NGS assay in CD138-negative samples. Healthy control samples were obtained from the Vanderbilt University Biobank, a de-identified biorepository with DNA samples [13]. We performed targeted, error-corrected NGS, using custom-designed probes for 22 CH-associated genes as described before (Supplementary Methods, Supplementary Fig. 1) [14]. Unique molecular identifiers (UMI) were used for error correction, excluding mutations detected from a single UMI. Comparisons were made using the chi-square or Fisher’s exact tests for categorical variables, and the Kruskal-Wallis test for continuous variables. The associations between CH mutations involving different genes and clinical variables were investigated by calculating the odds ratios with the Fisher exact test for categorical variables and the Wilcoxon rank-sum test for continuous variables.

**Fig. 1:**
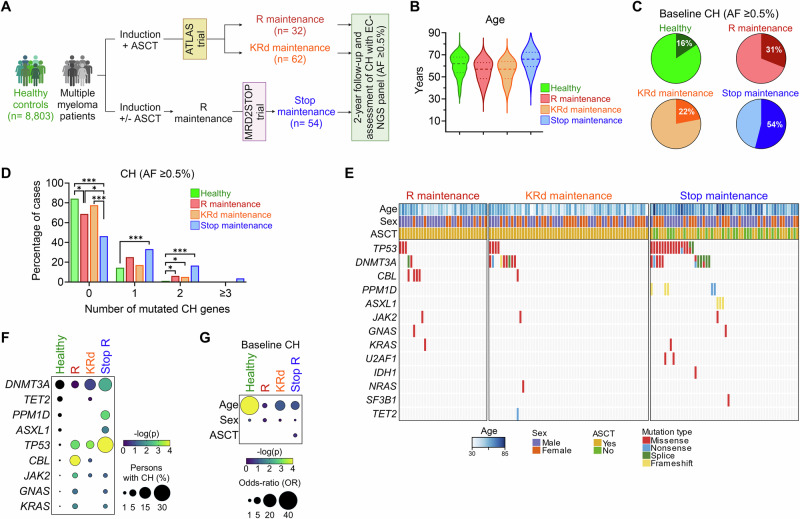
Clonal hematopoiesis (CH) is prevalent in patients with multiple myeloma (MM). **A** Schema summarizing healthy control and MM patient study cohorts. **B** Violin plots showing distribution of age between the four groups. **C** The frequency of CH in baseline samples from four groups. **D** Percentage of cases with detectable CH at baseline. **E** Oncoprint showing co-occurring and mutually exclusive mutations in three MM study groups. **F** Frequencies of specific CH gene mutations in four groups. **G** Odds-ratios for the associations between clinical variables and the presence of CH at baseline.

We studied 148 MM patients and 8803 age- and sex-matched healthy controls who were prospectively monitored for two years (Fig. 1A, B). To investigate the effects of two different maintenance therapies, we studied 94 patients with MM who received frontline induction therapy, ASCT, followed by maintenance therapy as part of the ATLAS trial with either R (*n* = 32) or KRd (*n* = 62). To study the effects of lenalidomide discontinuation, we examined a cohort of 54 MM patients who received frontline therapy followed by at least one year of lenalidomide maintenance and discontinued all therapy upon achieving ≤10^−6^ MRD negativity by IG-based NGS assay in MRD2STOP trial (Supplementary Table 1).

Using the traditional 2% AF cutoff, the incidence of CH was 7%, 6%, 7% and 30% in healthy control, pre-R maintenance, pre-KRd maintenance and pre-discontinuation (MRD2STOP) groups. Previous work showed that CH clones at AF <2% can progress during therapy and become founder mutations for subsequent t-MN and t-ALL [8, 15]. Therefore, we used an exploratory 0.5% cutoff, and the frequency of CH was low in healthy controls (16%), but higher in baseline samples from patients who were randomized to receive R maintenance (31%), KRd maintenance (22%) and those who stopped following maintenance (54%) (*p* < 0.001) (Fig. 1C). The higher CH frequency and the number of mutated CH genes pre-discontinuation in the MRD2STOP group may be attributed to their more extensive treatment history (Fig. 1D). When we compared the mutated genes between the groups, *TP53* mutations were more frequent in MM groups than healthy controls (Fig. 1E, F). When we investigated the association between clinical variables and baseline CH, older age correlated with higher CH frequency, but we did not identify sex-specific differences (Fig. 1G). Collectively, these data confirm the high frequency of *TP53-*mutant CH after induction and ASCT in patients with MM.

**Fig. 2:**
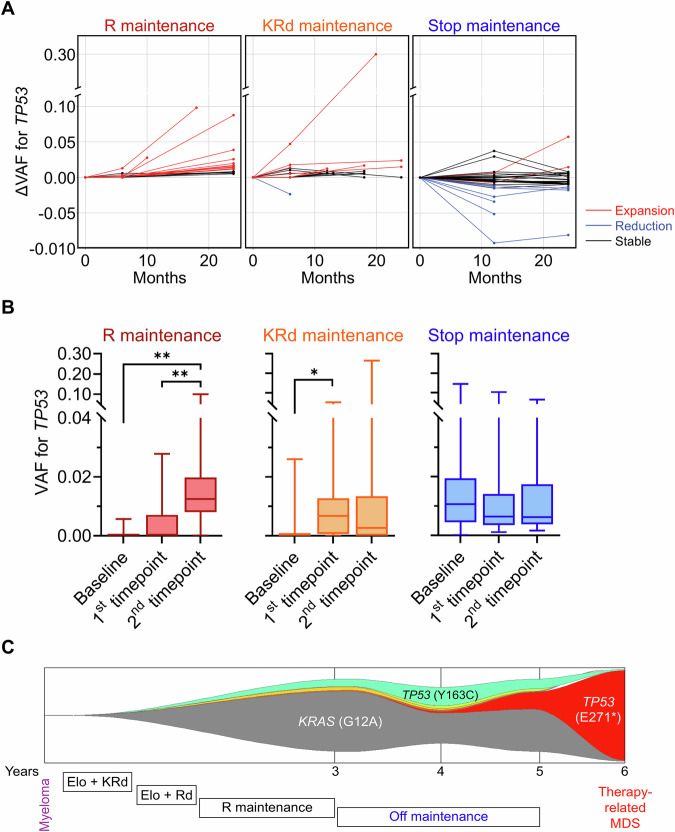
Evolution of clonal hematopoiesis (CH) with and without lenalidomide maintenance. **A** Plots demonstrating expansion, reduction or stability of *TP53-*mutant CH in three groups, measured by differences in variant allelic frequency (VAF) compared to baseline sample. **B** Box plots comparing VAF for *TP53* mutation at three different time points for three groups. **C** Fishplot showing progression of *TP53-*mutant CH (E271*) into therapy-related myelodysplastic syndrome.

Next, we investigated the evolution of CH in serial samples collected over two years (Supplementary Tables 2–4). In both R and KRd groups, we observed significant expansion of small *TP53-*mutant clones, which was more prominent in patients treated with lenalidomide maintenance alone (50% and 66%, respectively) (Fig. 2A, B). Among ATLAS trial patients with progressive *TP53-*mutant CH ( >1% increase in AF), two patients in the R maintenance arm progressed to t-AML. In the KRd arm, one progressed to t-MDS and two patients developed t-ALL. In contrast, we did not see significant changes in AF of age-related CH gene mutations (e.g., *DNMT3A*) in either treatment arm, suggesting selective expansion of *TP53-*mutant clones under lenalidomide (Supplementary Fig. 2A, B). Upon discontinuation of lenalidomide in the MRD2STOP group, *TP53-*mutant CH clones were stable in 53% of patients, while 29% of patients had regression of their *TP53-*mutant clones and only 18% had persistent clonal expansion. One patient with continued clonal expansion of *TP53-*mutant (E271*) CH despite lenalidomide discontinuation developed *TP53-*mutant therapy-related MN with complex karyotype three years after discontinuing lenalidomide (Fig. 2C). We confirmed that *TP53* and *KRAS* variants were not present in MM. Altogether, these data suggest that lenalidomide induces selective outgrowth of *TP53-*mutant CH, which can be a precursor for SPHM in patients with persistently progressive CH kinetics. Discontinuation of lenalidomide may halt this progression.

Lenalidomide maintenance prolonged survival in MM, though mainly in an era of less intensive induction. Since the evolution of induction therapy to quadruplets, along with new treatment options available for relapsed/refractory MM, the need for and the optimal duration of lenalidomide maintenance is being called into question. Long-term maintenance therapy is associated with increased risk for SPHM, therefore, an MRD-directed discontinuation strategy may help with risk reduction [12]. Our findings indicate that high-risk *TP53-*mutant CH is common in patients with MM prior to initiation of maintenance therapy. Continuous lenalidomide treatment selectively expands these clones. Clones at AF < 2% may still progress to leukemia with MM therapy. Clonal progression, which can be easily measured by serial monitoring, is a better predictor of future SPHM as opposed to cross-sectional analysis of CH at a given time point. We also showed, for the first time, that cessation of lenalidomide treatment may stabilize or regress high-risk CH. Therefore, prospective data obtained from the kinetics of CH may be combined with NGS MRD to identify patients who are at high risk for SPHM and can reduce the risk by discontinuing lenalidomide maintenance without compromising their MM response.

Our results can also guide future preventive interventions for individuals with CH. As the population of cancer survivors grows, the frequency of SPHM is expected to increase. There is an unmet need for meaningful surrogate endpoints in CH clinical trials. The gold-standard endpoint for CH clinical trials is hematologic malignancy, which may take years to decades to develop. Cross-sectional assessment of types and number of CH mutations, AF and hematologic parameters is not sufficient to accurately stratify SPHM risk. Based on our data in MM patients and published work from others, most individuals with high-risk CH (including *TP53-*mutant CH) may never progress to SPHM. In contrast, individuals with expanding CH, measured by serial AF assessment over two years, are at higher risk for SPHM. Therefore, dynamic CH assessment should be further validated as a potential biomarker that can serve as an endpoint for studies focusing on novel therapeutics for leukemia prevention.

## Supplementary information

Supplementary data

## Data Availability

Sequencing data are available in Supplementary Data. Additional requests on raw data can be directed to the Corresponding Author.
