# Supplementary material for: Evolution of clonal hematopoiesis on and off lenalidomide maintenance for multiple myeloma
Source: Leukemia. 2025 Jul 16;39(9):2285–8. doi: 10.1038/s41375-025-02707-2 (PMC12380616; doi:10.1038/s41375-025-02707-2)
Supplement: Supplementary file 1 — Supplementary data [file 41375_2025_2707_MOESM1_ESM.pdf]

Evolution of clonal hematopoiesis on and off lenalidomide maintenance for multiple myeloma
SUPPLEMENTARY DATA

Supplementary methods

We performed error-corrected next-generation sequencing (NGS) on serial peripheral blood samples from healthy controls and individuals participating in multiple myeloma trials (ATLAS, MRD2STOP), using a targeted 22-gene panel[1]. DNA was extracted using commercial extraction kits (Qiagen, Hilden, Germany). Our method was designed for high-throughput and cost-efficiency, compatible with the hybrid capture system leveraging the Twist Library Preparation Enzymatic Fragmentation Kit version 2.0 (cat# 104207; Twist Bioscience). Comparison of standard mechanical fragmentation and this enzymatic library preparation demonstrated highly concordant results. The hybrid capture technology was optimized to enrich DNA regions of interest from the pooled DNA libraries. With the custom-designed hybrid oligonucleotide probes, 12 times the number of hybridization reactions could be performed. The entire pipeline was established on a standard liquid handling robot platform (Biomek I7; Beckman Coulter, Brea, CA) to improve reproducibility. A specific set of DNA regions that encompass >95% of all clonal hematopoiesis mutations observed in the general population was identified, which covers the following target genes: *ASXL1, ASXL2, BRCC3, CBL, DNMT3A, ETNK1, GNAS, GNB1, IDH1, IDH2, JAK2, KIT, KRAS, MPL, NRAS, PPM1D, SETBP1, SF3B1, SRSF2, TET2, TP53,* and *U2AF1*. To increase the sensitivity of this assay, unique molecular identifiers (UMI) were used for error correction, excluding mutations detected from a single UMI. The mean coverage depth was 1,725x after de-duplication. Clonal hematopoiesis mutations were called for variants with ≥1000x total read depth, ≥3 variant allele reads, and >0.5% allelic frequency (AF) in at least one timepoint. Putative somatic mutations were identified in the aligned sequencing reads using the Mutect2 version 4.1.0.0–GATK version 4.1.4.1 software package, and filtering was applied to identify variants that met previously described criteria for clonal hematopoiesis[2].

For additional QC validation of variation in AF reporting, we ran technical replicates of individual samples **(Supplementary Figure 1)**. The standard deviation (SD) of AF reporting was 0.16% with range of 0.01% and 0.6%.

Supplementary Discussion

Currently available myeloid malignancy prediction tools (e.g., Clonal Hematopoiesis Risk Score[3]) were developed by studying the general population consisting mostly of healthy participants. Similarly, population-wide CH studies establishing cutoffs to define leukemic potentials of clones originated from biobanks of mostly healthy individuals[4]. These models and conclusions are not validated in solid tumor patients undergoing genotoxic therapy or MM patients receiving lenalidomide. Our findings provide important insights into the significance of clones at AF <2% for MM patients receiving lenalidomide therapy. While majority of these clones do not progress to leukemia, clones that expand >1% may lead to therapy-related AML or ALL in MM patients. Further research is needed to identify which of these small clones are truly pre-leukemic. Functional studies may decipher the leukemogenic potential of rising TP53 clones, or additional biomarkers (e.g., serum-based) may further stratify the risk for patients with progressive therapy-related CH. In addition, the number and diversity of

mutations may further modify this risk. Whether the presence of multiple small clones or different sizes of clones alter the competition between these clones should be further investigated.

The impact of different maintenance therapy approaches on second primary hematologic malignancy (SPHM) rates were examined in IFM2005, IFM2009 and DETERMINATION studies. In the IFM2005-02 study, patients were randomized to post-transplant lenalidomide vs placebo and the median duration of lenalidomide was 2 years[5]. The trial was stopped early due to the Data Safety Monitoring Board recommendation and concerns for SPHMs. The rates of SPHM were 2% vs 6.5% between placebo and lenalidomide groups, respectively. Despite the short duration of lenalidomide, high rates of SPHM on long-term follow-up suggest that lenalidomide-induced changes may not be fully reversible. In IFM2009 study, patients were randomized to either autologous stem cell transplant (ASCT) or extended bortezomib, lenalidomide, dexamethasone (VRd) without transplant[6]. Both arms received lenalidomide maintenance for 13 cycles. In the transplant arm, 7/350 patients developed SPHM (2%) as opposed to 5/350 patients in the non-ASCT arm (1.4%). Finally, in DETERMINATION study comparing ASCT vs no ASCT, but ongoing lenalidomide maintenance until progression, SPHM rates were 3.6% vs 2.5%, respectively[7]. Collectively, these data suggest that leukemogenic effect of lenalidomide may not be immediately reversible upon discontinuation of therapy. However, our data suggest that *TP53*-mutant CH clones may regress over time upon treatment cessation in some patients. Therefore, longer follow up is necessary to understand annual leukemia risk reduction upon discontinuation of therapy in MM patients who achieve MRD-negative remission.

Supplementary References

1. Mack T, Vlasschaert C, von Beck K, et al. Cost-Effective and Scalable Clonal Hematopoiesis Assay Provides Insight into Clonal Dynamics. *J Mol Diagn*. 2024;26(7):563-573.

2. Vlasschaert C, Mack T, Heimlich JB, et al. A practical approach to curate clonal hematopoiesis of indeterminate potential in human genetic data sets. *Blood*. 2023;141(18):2214-2223.

3. Weeks LD, Niroula A, Neuberg D, et al. Prediction of risk for myeloid malignancy in clonal hematopoiesis. *NEJM Evid*. 2023;2(5).

4. Jaiswal S, Fontanillas P, Flannick J, et al. Age-related clonal hematopoiesis associated with adverse outcomes. *N Engl J Med*. 2014;371(26):2488-2498.

5. Attal M, Lauwers-Cances V, Marit G, et al. Lenalidomide maintenance after stem-cell transplantation for multiple myeloma. *N Engl J Med*. 2012;366(19):1782-1791.

6. Attal M, Lauwers-Cances V, Hulin C, et al. Lenalidomide, Bortezomib, and Dexamethasone with Transplantation for Myeloma. *N Engl J Med*. 2017;376(14):1311-1320.

7. Richardson PG, Jacobus SJ, Weller EA, et al. Triplet Therapy, Transplantation, and Maintenance until Progression in Myeloma. *N Engl J Med*. 2022;387(2):132-147.

SUPPLEMENTARY FIGURES

Supplementary Figure 1

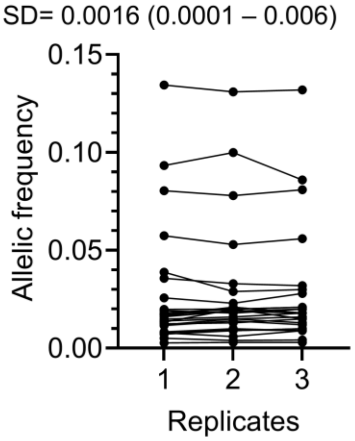

Plot demonstrating replicate allelic frequency measurements of same samples over three times.

Supplementary Figure 2

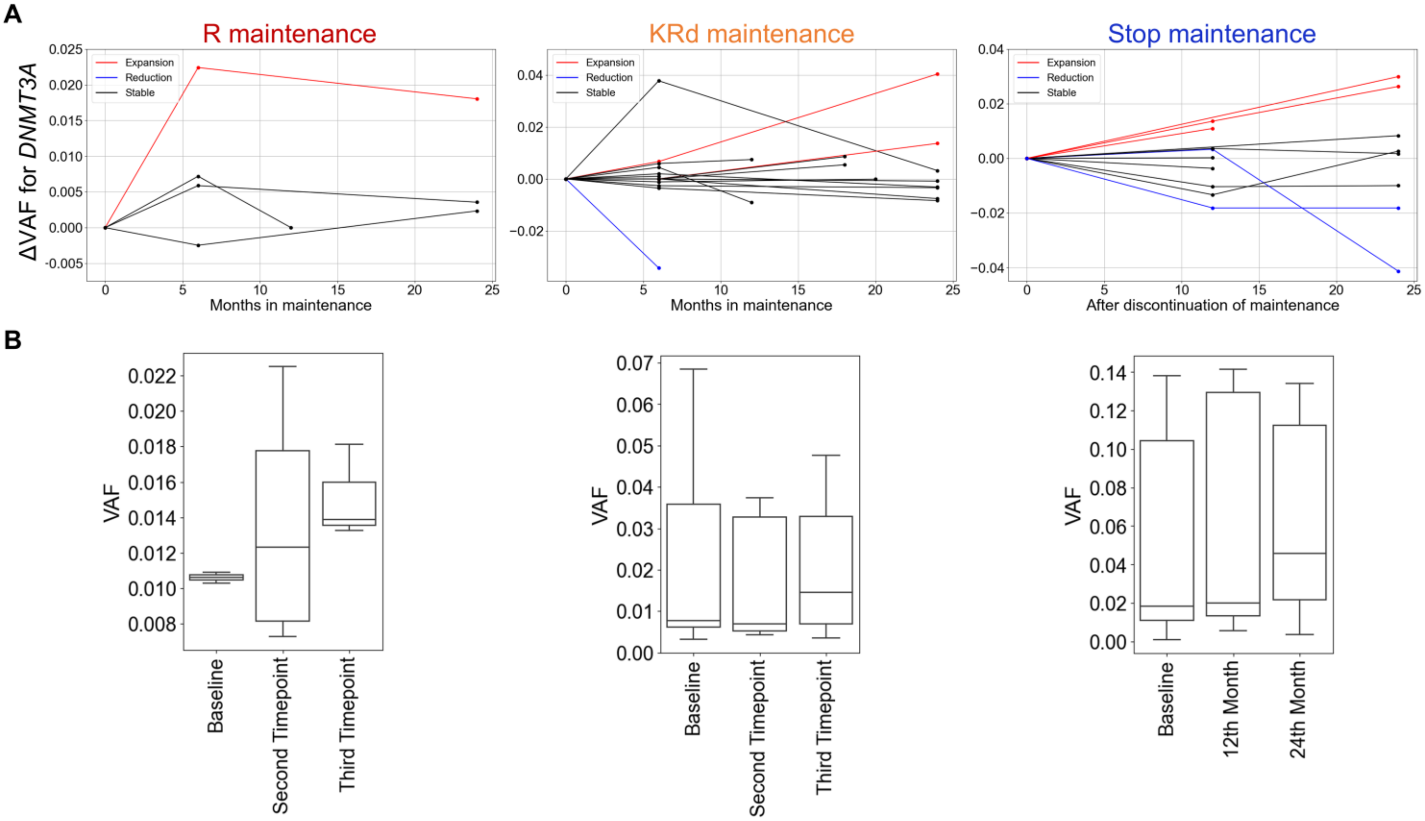

(A) Plots demonstrating expansion, reduction or stability of *DNMT3A*-mutant CH in three groups, measured by differences in variant allelic frequency (VAF) compared to baseline sample. **(B)** Box plots comparing VAF for *DNMT3A* mutation at three different time points for three groups.

SUPPLEMENTARY TABLES

Supplementary Table 1. Study cohort

	**Healthy controls (n= 8,803)**	**R maintenance (n= 32)**	**KRd maintenance (n= 62)**	**Stop maintenance (n=54)**
Age, median (years)	62 (32 – 85)	57 (35 – 69)	57 (32 – 70)	66 (39 – 84)
**Gender, n (%)**				
Male	4475 (50.8)	23 (71.9)	30 (48.4)	32 (59.2)
Female	4328 (49.2)	9 (28.1)	32 (51.6)	22 (40.8)
**Ethnicity, n (%)**				
White	7143 (81.1)	31 (96.9)	58 (93.6)	40 (74.2)
Black	1456 (16.5)	0	3 (4.8)	12 (22.2)
Asian	67 (0.8)	0	0	1 (1.8)
Other	16 (0.2)	0	0	1 (1.8)
Unknown	121 (1.4)	1 (3.1)	1 (1.6)	0
**Induction therapy, n (%)**				
Doublet	NA	1 (3.1)	1 (1.6)	1 (1.8)
Triplet		31 (96.9)	61 (98.4)	23 (42.6)
Quadruplet		0	0	26 (48.2)
Other		0	0	4 (7.4)
**Autologous stem cell transplant, n (%)**	NA	32 (100)	62 (100)	31 (57.4)
**Duration of consolidation and maintenance therapy, months, median (range)**	NA	33 (2 – 53)	33 (5 – 62)	35 (12 – 95)
**Persons with CH, n (%)**	1,381 (15.7)	10 (31.2)	13 (22.4)	29 (53.7)
DNMT3A	775 (3.5)	2 (6.3)	8 (13.8)	11 (20.4)
TET2	305 (3.5)	0	1 (1.7)	0
PPM1D	134 (1.5)	0	0	5 (9.3)
ASXL1	108 (1.2)	0	0	3 (5.6)
TP53	55 (0.6)	3 (9.4)	4 (6.9)	16 (29.6)
CBL	14 (0.2)	4 (12.5)	1 (1.7)	0

Supplementary Table 2. Allelic frequencies (AF) for pathogenic/likely pathogenic variants in R maintenance group

PatientID	Gene	cDNA change	Protein Change	0	6 mo	18 mo	21 mo	24 mo
01-39	*TP53*	c.542G>A	Arg181His	0.0020	0.0078			0.0078
01-39	*ASXL1*	c.1934dup	Gly646TrpfsTer12					0.0804
01-25	*TP53*	c.993+1G>A						0.0257
01-25	*TP53*	c.743G>A	Arg248Gln					0.0388
01-25	*TP53*	c.469G>T	Val157Phe					0.0051
02-12	*CBL*	c.1247G>C	Cys416Ser	0.0051	0.0086			0.0027
02-12	*GNAS*	c.2531G>A	Arg844His	0.0025	0.0774			0.0574
01-57	*CBL*	c.1139T>C	Leu380Pro	0.0039			0.0051	
01-57	*PPM1D*	c.1281G>A	Trp427Ter				0.0122	
01-32	*ASXL1*	c.1934dup	Gly646TrpfsTer12					0.1344
01-37	*TP53*	c.406C>G	Gln136Glu		0.0020			0.0081
02-06	*TP53*	c.488A>G	Tyr163Cys	0.0011	0.0139	0.0992		
01-73	*CBL*	c.1222T>C	Trp408Arg	0.0147	0.0154	0.0124		
01-09	*KRAS*	c.34G>C	Gly12Arg	0.0049	0.0079			
01-11	*SF3B1*	c.1998G>C	Lys666Asn					0.0357
01-11	*KIT*	c.2466T>A	Asn822Lys					0.0192
01-11	*TP53*	c.988del	Leu330PhefsTer15					0.0161
01-11	*TP53*	c.786del	Asn263IlefsTer82					0.0125
01-11	*TP53*	c.740A>G	Asn247Ser					0.0072
01-11	*TP53*	c.700T>A	Tyr234Asn					0.0198
01-11	*TP53*	c.537T>A	His179Gln	0.0057	0.0071			0.0933
01-11	*TP53*	c.524G>A	Arg175His					0.0116
01-11	*TP53*	c.503A>T	His168Leu					0.0120
01-11	*TP53*	c.482C>A	Ala161Asp					0.0148
01-11	*TP53*	c.451C>T	Pro151Ser	0.0036	0.0072			0.0173
01-11	*TP53*	c.422G>T	Cys141Phe					0.0082
01-11	*TP53*	c.421T>A	Cys141Ser					0.0171
01-11	*TP53*	c.159G>A	Trp53Ter					0.0083
01-10	*DNMT3A*	c.2322+1G>A		0.0109	0.0084			0.0133
01-10	*CBL*	c.1259G>A	Arg420Gln	0.0293	0.0103			0.0163
01-36	*DNMT3A*	c.1238dup	Phe414LeufsTer7		0.0225			0.0181
01-34	*DNMT3A*	c.920C>T	Pro307Leu	0.0103	0.0162			0.0139
02-01	*DNMT3A*	c.1554+1G>A			0.0073			
01-61	*JAK2*	c.1849G>T	Val617Phe	0.0060				
07-07	*TP53*	c.375+1G>C			0.0278			

Supplementary Table 3. Allelic frequencies (AF) for pathogenic/likely pathogenic variants in KRd maintenance group

Patient ID	Gene	cDNA change	Protein Change	0	6 mo	12 mo	18 mo	20 mo	24 mo
18629	*TP53*	c.376T>C	Tyr126His		0.0103				
16803	*TP53*	c.1024C>T	Arg342Ter		0.0128				
16803	*NRAS*	c.52G>A	Ala18Thr		0.0217				
45686	*TET2*	c.2248C>T	Gln750Ter	0.0028	0.0158				0.0053
45686	*CBL*	c.1111T>C	Tyr371His	0.0022	0.0064				0.0028
22647	*DNMT3A*	c.920C>T	Pro307Leu	0.0330	0.0374	0.0239			
45674	*DNMT3A*	c.745C>T	Gln249Ter	0.0063	0.0051			0.0062	
45674	*TP53*	c.659A>G	Tyr220Cys	0.0036	0.0506			0.2646	
25934	*ASXL1*	c.1934dup	Gly646TrpfsTer12		0.0382		0.0903		
27395	*DNMT3A*	c.1676G>A	Cys559Tyr				0.0055		
45683	*DNMT3A*	c.2644C>T	Arg882Cys		0.0068				0.0405
45683	*TP53*	c.380C>G	Ser127Cys	0.0016	0.0192				0.0253
15707	*TP53*	c.659A>G	Tyr220Cys	0.0011	0.0069	0.0071			
45680	*DNMT3A*	c.2086C>T	Gln696Ter		0.0060	0.0076			
45858	*KIT*	c.1670G>A	Trp557Ter		0.0054				
45779	*DNMT3A*	c.2644C>T	Arg882Cys	0.0444	0.0822				0.0476
14	*DNMT3A*	c.2312G>A	Arg771Gln		0.0153				0.0145
14	*DNMT3A*	c.1627G>A	Gly543Ser		0.0327				0.0252
14	*DNMT3A*	c.1554+1G>A			0.0285				0.0422
24473	*DNMT3A*	c.2479-1G>A		0.0060	0.0070				
20455	*JAK2*	c.1849G>T	Val617Phe	0.0157	0.0119				0.0021
1	*TP53*	c.472C>G	Arg158Gly		0.0139				
45812	*DNMT3A*	c.1374del	Lys459ArgfsTer192	0.0032	0.0052				
45840	*TP53*	c.742C>T	Arg248Trp			0.0057			
45840	*TP53*	c.724T>C	Cys242Arg			0.0123			
45840	*TP53*	c.575del	Gln192ArgfsTer55			0.0089			
45840	*TP53*	c.377A>G	Tyr126Cys			0.0065			
45840	*KRAS*	c.40G>A	Val14Ile			0.0065			
23377	*PPM1D*	c.1535del	Asn512IlefsTer2				0.0061		
45687	*DNMT3A*	c.920C>T	Pro307Leu	0.0069	0.0043				0.0035
45853	*DNMT3A*	c.2645G>A	Arg882His	0.0685	0.0342				
45853	*TP53*	c.641A>G	His214Arg	0.0260	0.0023				
45855	*TP53*	c.919+1G>T			0.0051				0.0149
16438	*DNMT3A*	c.2644C>T	Arg882Cys	0.0084	0.0049				
14611	*NRAS*	c.35G>A	Gly12Asp	0.0212					
45839	*PPM1D*	c.1535del	Asn512IlefsTer2		0.0057				
45691	*DNMT3A*	c.2177G>T	Gly726Val				0.0087		
45691	*TP53*	c.919+1G>A					0.0060		
45691	*TP53*	c.814G>A	Val272Met				0.0052		
45691	*TP53*	c.733G>A	Gly245Ser				0.0088		
45691	*TP53*	c.646G>A	Val216Met				0.0167		

Supplementary Table 4. Allelic frequencies (AF) for pathogenic/likely pathogenic variants in stop maintenance (MRD2STOP) group

PatientID	Gene	cDNA change	Protein Change	0	12 mo	24 mo
M52	*DNMT3A*	c.1667+1G>A		0.0091		
M27	*TP53*	c.818G>A	Arg273His	0.0026	0.0029	0.0085
M27	*TP53*	c.814G>A	Val272Met	0.0040		0.0070
M27	*TP53*	c.734G>A	Gly245Asp	0.0075	0.0017	
M27	*TP53*	c.711G>A	Met237Ile	0.0033	0.0050	
M27	*TP53*	c.427G>A	Val143Met	0.0023	0.0077	0.0049
M31	*DNMT3A*	c.2645G>A	Arg882His	0.1043		0.1342
M31	*DNMT3A*	c.958C>T	Arg320Ter	0.0375		0.0458
M31	*IDH1*	c.395G>A	Arg132His	0.0618		0.0894
M23	*DNMT3A*	c.1627G>A	Gly543Ser	0.0156	0.0119	
M23	*DNMT3A*	c.1554+1G>A		0.0199	0.0201	
M42	*ASXL1*	c.1900_1922del	Glu635ArgfsTer15	0.0049	0.0057	
M20	*DNMT3A*	c.2409-1G>A		0.1380	0.1277	0.1281
M16	*TP53*	c.814G>A	Val272Met		0.0031	0.0077
M18	*DNMT3A*	c.1792C>T	Arg598Ter	0.1383	0.1416	0.0969
M18	*TP53*	c.427G>A	Val143Met	0.0234	0.0162	0.0381
M18	*TP53*	c.396G>C	Lys132Asn	0.0365	0.0095	0.0235
M11	*DNMT3A*	c.2479-1G>A		0.0134		0.0161
M14	*KRAS*	c.34G>C	Gly12Arg	0.2034	0.0968	0.1376
M14	*TP53*	c.817C>T	Arg273Cys	0.0039	0.0054	0.0016
M14	*TP53*	c.811G>T	Glu271Ter		0.0067	0.0575
M14	*TP53*	c.711G>A	Met237Ile	0.0112	0.0055	0.0027
M14	*TP53*	c.524G>A	Arg175His	0.0020	0.0100	0.0080
M14	*TP53*	c.488A>G	Tyr163Cys	0.0242	0.0617	0.0323
M14	*TP53*	c.826G>C	Ala276Pro	0.0089	0.0063	
M39	*DNMT3A*	c.2645G>A	Arg882His	0.0011	0.0146	0.0274
M39	*TP53*	c.530C>T	Pro177Leu	0.0229	0.0201	0.0153
M3	*TP53*	c.731G>T	Gly244Val	0.0128	0.0029	0.0040
M3	*TP53*	c.578A>G	His193Arg	0.1450	0.0529	0.0642
M3	*TP53*	c.472C>T	Arg158Cys	0.0077	0.0025	0.0025
M3	*TP53*	c.158G>A	Trp53Ter	0.0042	0.0338	0.0047
M38	*TET2*	c.885del	Asn296IlefsTer18		0.0070	
M38	*PPM1D*	c.1654C>T	Arg552Ter	0.0057		
M29	*TP53*	c.830G>A	Cys277Tyr	0.0638	0.0125	
M29	*TP53*	c.672+1G>A		0.0251	0.0134	
M29	*TP53*	c.421T>C	Cys141Arg	0.0141	0.0011	
M29	*TP53*	c.919+2T>A		0.0062		
M34	*DNMT3A*	c.2204A>G	Tyr735Cys	0.0019	0.0055	0.0036
M34	*TP53*	c.376T>G	Tyr126Asp	0.0150		0.0054
M34	*TET2*	c.1651C>T	Gln551Ter			0.0073
M10	*JAK2*	c.1849G>T	Val617Phe	0.0147	0.1411	0.2455
M10	*ASXL1*	c.1934dup	Gly646TrpfsTer12	0.1277	0.0763	
M13	*ASXL1*	c.1900_1922del	Glu635ArgfsTer15		0.0077	0.0036
M13	*ASXL1*	c.1934dup	Gly646TrpfsTer12	0.2367	0.2166	0.2771
M45	*TP53*	c.818G>A	Arg273His	0.0167		

M45	*TP53*	c.814G>A	Val272Met	0.0070		
M45	*TP53*	c.488A>G	Tyr163Cys	0.0109		
M45	*PPM1D*	c.1637del	Leu546ArgfsTer2	0.0825		
M37	*DNMT3A*	c.2645G>A	Arg882His	0.1203	0.1312	
M37	*TP53*	c.818G>A	Arg273His	0.1370	0.1032	
M37	*TP53*	c.517G>A	Val173Met	0.0221	0.0162	
M41	*GNAS*	c.2531G>A	Arg844His	0.0032	0.0070	
M26	*DNMT3A*	c.2479-1G>A		0.0183		
M43	*PPM1D*	c.1714C>T	Arg572Ter	0.0103	0.0048	
M9	*TP53*	c.783-2A>G		0.0153	0.0040	
M22	*TP53*	c.844C>T	Arg282Trp	0.0139	0.0088	0.0052
M22	*TP53*	c.736A>G	Met246Val	0.0209	0.0063	0.0033
M22	*TP53*	c.584T>C	Ile195Thr	0.0118	0.0065	0.0021
M22	*TP53*	c.713G>A	Cys238Tyr	0.0058		
M25	*TP53*	c.743G>A	Arg248Gln			0.0052
M25	*TP53*	c.659A>G	Tyr220Cys	0.0061		
M49	*DNMT3A*	c.2644C>T	Arg882Cys	0.0109		
M49	*TP53*	c.711G>A	Met237Ile	0.0190		
M49	*TP53*	c.646G>A	Val216Met	0.0180		
M49	*PPM1D*	c.1637del	Leu546ArgfsTer2	0.0093		
M48	*TP53*	c.584T>C	Ile195Thr	0.0072		
M48	*PPM1D*	c.1636dup	Leu546ProfsTer6	0.0127		
M48	*U2AF1*	c.101C>T	Ser34Phe	0.0912		
M32	*SF3B1*	c.1998G>T	Lys666Asn	0.1525	0.0669	0.0359
M1	*TP53*	c.517G>A	Val173Met	0.0046	0.0036	0.0100
M44	*TP53*	c.734G>A	Gly245Asp	0.0103	0.0058	
M44	*TP53*	c.427G>A	Val143Met	0.0052	0.0016	
M44	*U2AF1*	c.101C>T	Ser34Phe	0.3604	0.2891	
